# Effects of Arabidopsis wall associated kinase mutations on ESMERALDA1 and elicitor induced ROS

**DOI:** 10.1371/journal.pone.0251922

**Published:** 2021-05-20

**Authors:** Bruce D. Kohorn, Bridgid E. Greed, Gregory Mouille, Stéphane Verger, Susan L. Kohorn

**Affiliations:** 1 Department of Biology, Bowdoin College, Brunswick, Maine, United States of America; 2 IJPB, INRAE, AgroParisTech, Université Paris-Saclay, RD10, Versailles Cedex, France; 3 Department of Forest Genetics and Plant Physiology, Umeå Plant Science Centre, Swedish University of Agricultural Sciences, Umeå, Sweden; Iowa State University, UNITED STATES

## Abstract

Angiosperm cell adhesion is dependent on interactions between pectin polysaccharides which make up a significant portion of the plant cell wall. Cell adhesion in Arabidopsis may also be regulated through a pectin-related signaling cascade mediated by a putative O-fucosyltransferase ESMERALDA1 (ESMD1), and the Epidermal Growth Factor (EGF) domains of the pectin binding Wall associated Kinases (WAKs) are a primary candidate substrate for ESMD1 activity. Genetic interactions between WAKs and ESMD1 were examined using a dominant hyperactive allele of WAK2, *WAK2cTAP*, and a mutant of the putative O-fucosyltransferase ESMD1. WAK2cTAP expression results in a dwarf phenotype and activation of the stress response and reactive oxygen species (ROS) production, while *esmd1* is a suppressor of a pectin deficiency induced loss of adhesion. Here we find that *esmd1* suppresses the WAK2cTAP dwarf and stress response phenotype, including ROS accumulation and gene expression. Additional analysis suggests that mutations of the potential WAK EGF O-fucosylation site also abate the WAK2cTAP phenotype, yet only evidence for an N-linked but not O-linked sugar addition can be found. Moreover, a *WAK* locus deletion allele has no effect on the ability of *esmd1* to suppress an adhesion deficiency, indicating WAKs and their modification are not a required component of the potential ESMD1 signaling mechanism involved in the control of cell adhesion. The WAK locus deletion does however affect the induction of ROS but not the transcriptional response induced by the elicitors Flagellin, Chitin and oligogalacturonides (OGs).

## Introduction

Cell adhesion in plants is dependent on pectin polysaccharides which comprise a major portion of the immediate interface between cells [1, 2]. The cell wall is initially deposited at the cell plate during cell division, and through a combination of cellulose secretion at the plasma membrane, and secretion through the endomembrane system of pectin and hemicellulose and other polysaccharides, a primary wall is elaborated [3–7]. Enzymatic activity that further modifies the polymers can have dramatic effects on the rigidity and elasticity of the cell wall. For example, the de-esterification of pectin mediated by pectin methylesterase (PME) [8], and inhibited by PME inhibitors (PMEI) regulate the charge and calcium dependent crosslinking and are associated with increased or decreased wall extensibility and adhesion [9–14]. Polygalacturonases and pectate lyases cleave pectin, but their activity can be influenced by the degree of pectin esterification, resulting in a complex interplay of enzyme expression profiles and substrate/enzyme pairing across different tissue types and plant species [15].

Mutations that either reduce or modify the pectin content or change their modification can cause a loss of cell adhesion. Mutations in *QUASIMODO 1 and 2* (*QUA1*,*2*) that encode a Golgi-localized glycosyl and methyl transferase, respectively, cause a 50% reduction in pectin and a significant loss of cell adhesion most easily detected in expanding hypocotyls [16–20]. Yet mutations in *FRIABLE* 1 (*FRB1)*, that also induces a similar adhesion defect, change the amount of galactose and arabinose containing oligosaccharides in the Golgi, and alters pectin methyl esterification and xyloglucan microstructure, but do not change the total pectin amount [21]. But cell adhesion in Arabidopsis may also be regulated by a signaling cascade mediated by the putative O-fucosytransferase ESMERALDA1 (ESMD1), which by sequence similarity to metazoan enzymes is thought to use an Epidermal Growth Factor (EGF) domain as a substrate for the addition of a single fucose to a serine or threonine at the consensus C_2_XXXXS/TC_3_ (where X is any amino acid and numbers indicate one of six Cysteines, S1 Fig) [22]. EGF domains are characterized by a series of 6 repeated and regularly spaced Cysteines, and their fucosylation can lead to alteration of receptor activity in metazoans [23–26]. Mutations in *ESMD1* can suppress *qua2-1* and *frb1* yet there is no restoration of pectin levels [22]. The nature of this putative signaling pathway is not known, but clues to its identity may lie in the plant receptor kinases that contain an EGF domain.

At least two families of receptor proteins in Arabidopsis contain EGF domains with a potential O-fucosylation site [22]. The family of 6, G-type lectin S-receptor-like serine/threonine-protein kinase each have one EGF domain with a O-fucosylation consensus sequence. However, no reports to date link these type of receptors to the cell wall or pectin. But the pectin binding Wall associated Kinases (WAKs) also contain a potential ESMD1 substrate and since there is ample evidence that WAKs are involved in pectin signaling [27–32], they are a primary candidate for ESMD1 mediated O-fucosylation. WAKs are receptor-like protein kinases with several extracellular EGF domains, and a cytoplasmic serine/threonine kinase. WAKs bind to native cell wall pectin and are required for cell expansion [28], but they also bind to pectin fragments or oligogalacturonides (OGs) that may be generated during wounding or exposure to pathogen, dark/light and induce a stress response [30, 32–34]. OGs of shorter lengths are also involved in photomorphogenesis [35]. How WAKs distinguish these OGs from native pectin is not known, but it has been suggested that long polymers and fragmented pectin compete for WAK activation to stimulate alternate pathways [27].

To explore whether WAKs are involved in a potential ESMD1 dependent signaling pathway that affects cell adhesion, interactions between WAKs and ESMD1 were examined using several alleles of *WAK* and *ESMD1*. Results support a role for the modification of the EGF WAK domain in WAK signaling but not in *esmd1* suppression of adhesion defects. A new 25 Kb deletion was created in the locus that contains the 5 WAKs and this revealed that WAKs play a role in the ROS response to multiple elicitors.

## Materials and methods

### Plant growth conditions

*Arabidopsis thaliana* seeds were sterilized for 5 minutes in 95% ethanol and then 5 minutes in 10% bleach and rinsed twice with sterile dH_2_O. Seeds were then planted on agar containing Murashige and Skoog (MS) media (Sigma Aldrich) pH 5.7 with 2% agarose and 1% sucrose or planted directly onto soil. Following planting, seeds were exposed to cold (4°C) for 48 hours, and grown at 20°C in 8 hrs of dark, 16 hrs of light. For in-experiment comparisons, samples were grown at the same time in six replicates. Plants were imaged using a Nikon D3000 camera or Leica DM350 with a Wild dissecting microscope. Total leaf area of each plant was measured using ImageJ.

### DNA extraction and PCR

Three week-old healthy green leaves from plants of interest were collected, frozen in liquid N_2,_ and DNA was extracted as described [36]. The indicated genes were PCR amplified according to the manufacturer’s conditions using Titanium Taq DNA polymerase (Takara Bio, Mnt View CA), or for long range PCR Platinum Superfi DNA polymerase (In Vitrogen/Thermo Fisher Waltham MA) using primers shown in S2A and S2C Fig. *FADlox* RT-qPCR was performed as described on biological triplicates [31, 36].

### Western blotting was performed as described [30]

*ROS Assay* was performed on 6 biological replicates essentially as described [37] using a GloMax^®^ 96-well microplate luminometer and results were analyzed using Prism. Samples were recorded every minute for 60 minutes, and the area under the curve was calculated using Prism.

*Ruthenium Red* Staining was performed according to established protocols [22]. All seedlings were incubated in MS media 1% sucrose at 4°C for 2 days, then 4h in light prior to be transferred for 4 days in the dark at 22° C before staining for 2 minutes in 0.5 mg/ml Ruthenium Red (Sigma Corp. St. Louis) in water.

#### CRISPR

The WAK4 and WAK2 oligonucleotides used as templates for SgRNAs (S3A Fig) were each cloned into pSkAtu26 as described [38], and inserted into pCambia 1302 that also had a pOLE1-RFP cassette inserted into the ASN718 site by PCR cloning [39].

*Glycosidase* reactions were according to the manufacturer (NEB Ipswich, MA). Three week old leaves were ground in 50 mM NaPO_4_ pH 7.5, 40 mM DTT, 0.5% DTT and protease inhibitor cocktail (Sigma Corp. St Louise MO), heated at 95°C for 5 min. and then centrifuged at 10,000xg for 10 minutes. The supernatant of 0.1 mg/ml protein was divided into equal aliquots and adjusted for each treatment- NO; 2.5 units neuraminidase and 2,000 units O-glycosidase, 1% NP40. NF; 25 units peptide N-glycosidase F, 1% NP40. FU; 0.2 u/μL α1–3,4 Fucosidase, 5 mM CaCl_2_, 50 mM NaAcetate pH 5.5. Samples were incubated at 37° C for 60 min, and then 95° C in denaturing Laemmli buffer and run in a denaturing polyacrylamide gel and Western blotted for the TAP tag [30].

*RNA seq* and bioinformatics was performed by Novogen Corp. (Sacramento Ca.) on biological triplicate, 3 week old leaf RNA samples isolated using a Qiagen RNA isolation kit (Germantown MD.) Analysis of specific transcripts was carried out using Novogene BAM files and the IGV program from the Broad Inst. (Cambridge MA)

#### Elicitor treatment

OG s degree of polymerization (dp) 9–15 were prepared according to [30]. Seeds were plated in a 5 ml well of a plastic dish in 0.5X MS medium plus vitamins, vernalized for 3 days, and incubated at 22°C with gentle shaking under 24-h light. After 7 days at 22°C, OGs were added to a final concentration of 50 μg/ml, Flg22 to 10 μg/ml, or a Chitin suspension in dH_2_0 to 1mg/ml, or mock treatment of dH_2_O, and shaken for an additional 3 hr. Then seedlings were frozen in liquid nitrogen. Experiments were done in biological triplicates.

## Results

### *esmd1-1* suppresses *WAK2cTAP*

**Fig 1:**
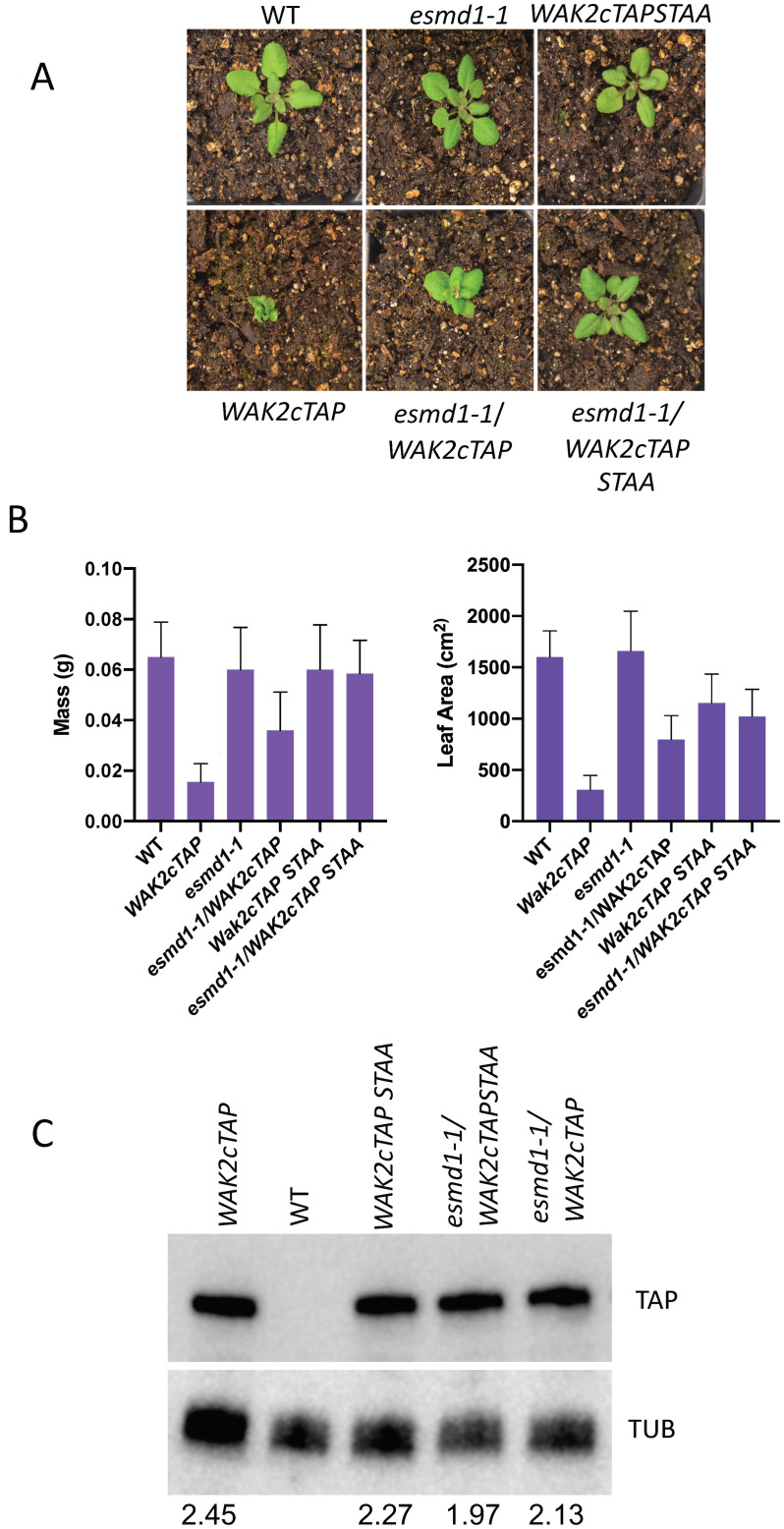
*esmd1-1* suppresses WAK2cTAP. A) Representative plants of indicated genotype grown under the same conditions. B) Quantification of wet mass and total leaf area of plants (n = 6) of indicated genotype. An ANOVA (Area; F(5,28) = 18.87 p<0.0001. Mass; F (5, 34) = 14.63 p<0.0001) indicates a difference between the samples, and Tukey’s tests for individual sample comparisons are shown in S2 Fig. Error bars indicate standard deviation. C) Western blot analysis of total protein extracts from plants of the indicated genotype indicating equivalent WAK2cTAP expression (TAP top) relative to tubulin loading control (TUB bottom). The ratio of the tubulin to WAK2cTAP from 3 samples is indicated below the Westerns and ANOVA and Tukey’ tests find no significant differences (S2 Fig).

ESMD1 has been proposed to fucosylate the EGF domain of WAKs based upon a) the presence of a conserved O-fucosylation motif in WAKs (S1 Fig) and S1b) the observation that both ESMD1 and WAKs are involved in pectin regulation [22]. To explore possible interactions between ESMD and WAKs a double mutant of a hyperactive *WAK2cTAP* allele and a loss of function *esmd1-1* were generated. *esmd1-1* was isolated as a suppressor of a pectin and adhesion deficient *qua2-1* mutant [22], and *WAK2cTAP* is a dominant hyperactive kinase allele that causes a constitutive stress response and dwarfism [31]. Strong loss of function alleles of *WAK*s have not been identified except for antisense WAK constructs that likely target more than the WAKs [40]. In addition, to assess the importance of the conserved fucosylation site, one of the potential WAK fucosylation sites was mutated by replacing serine (S) and threonine (T) residues at amino acid position 21 and 22 in the EGF domain with alanine (A) residues (*WAK2cTAP STAA*, green letters S1 Fig). Homozygous *esmd1-1* plants were crossed to the homozygous *WAK2cTAP* line and to the *WAK2cTAP STAA* line and resulting F_2_ progeny were screened for homozygous alleles of *WAK2cTAP (STAA)* and *esmd1-1*. WAK2cTAP (STAA) was detected by Western blot and plants with homozygous insertions (2 copies) were identified by segregation of the linked Basta resistant marker. *esmd1-1* was detected by sequencing the *ESMD1* PCR products from each plant. Soil grown, *esmd1-1*^-/-^*WAK2cTAP* and *esmd1-1*^-/-^*WAK2cTAP STAA* double mutants were subject to analysis by visual phenotype, wet weight, and leaf area. Consistent with previous findings, expression of the dominant active WAK2cTAP resulted in the characteristic stressed and dwarf phenotype, displaying curly leaves and signs of necrosis in comparison to WT plants [30, 31] (Fig 1A). The homozygous *esmd1-1* single mutant displays no obvious size related phenotype, resembling its WT counterpart (Fig 1A). However, the *esmd1-1*^-/-^*WAK2cTAP* results in an intermediate sized phenotype that is larger than the WAK2cTAP phenotype, but smaller than the *esmd1-1*^-/-^ (Fig 1A). This intermediate phenotype indicates that *esmd1-1* partially suppresses the dwarf, hyperactive *WAK2cTAP* phenotype. The mass and total leaf area of each plant was measured to quantify the observed size differences and an ANOVA indicated there was a difference between the samples (Area; F(5,28) = 18.87 p<0.0001. Mass; F (5, 34) = 14.63 p<0.0001). Tukey’s tests between each sample (S2 Fig) indicate that there is a difference (p<0.0001) between *WAK2cTAP* and *esmd1-1*^-/-^*WAK2cTAP* (Fig 1B). Additionally, *WAK2cTAP* and *esmd1-1*^-/-^*WAK2cTAP* were different in both mass and leaf area measurements from WT respectively, while *esmd1-1*^-/-^ was not. However, *esmd1-1*^-/-^ and *esmd1-1*^-/-^*WAK2cTAP* were only different in leaf area and not in mass, further suggesting that *esmd1-1*^-/-^ only partially suppresses *WAK2cTAP*.

Since the extracellular domain of WAK2cTAP is required for its activity [31] the STAA mutation was expected to affect the WAK2cTAP dominant phenotype if fucosylation plays a role. Fig 1A shows that plants expressing the WAK2cTAP allele are dwarf, while those expressing WAK2cTAP STAA are larger and similar in size to WT plants (Fig 1A and 1B). The *esmd1-1*^-/-^*WAK2cTAP STAA* double mutant is of a similar size to WAK2cTAP STAA as expected (Fig 1A and 1B). Measurements followed by ANOVA with Tukey’s test analysis of mass and leaf area confirm that there is a difference between WAK2cTAP and WAK2cTAP STAA or *esmd1-1*^-/-^*WAK2cTAP STAA* plants, respectively (Area; F(5,28) = 18.87 p<0.0001. Mass; F (5, 34) = 14.63 p<0.0001). Tukey’s tests for individual sample comparisons are shown in S2 Fig. Additionally, there is no difference between either the mass or leaf area of WAK2cTAP STAA and *esmd1-1*^-/-^*WAK2cTAP STAA* (Tukey’s test S2 Fig) as expected since both *esmd1-1* and STAA partially suppress WAK2cTAP. There is also no difference in either mass or leaf area between *esmd1-1*^-/-^*WAK2cTAP STAA* and *esmd1-1*^-/-^*WAK2cTAP*, confirming partial suppression in both cases.

To confirm that the partial suppression of the WAK2cTAP by *esmd1-1*, and the loss of the dwarf phenotype in *WAK2cTAP STAA* is not a result of differing levels of WAK2cTAP expression, equal total protein extracts of WAK2cTAP and *esmd1-1*^-/-^*WAK2cTAP and WAK2cTAP STAA* mutant lines were blotted to detect WAK2cTAP protein and were found to display similar protein levels, relative to the tubulin standard (Fig 1C). These results show that *esmd1-1* partially suppresses the *WAK2cTAP* phenotype and suggest that ESMD1 activity is required for the WAK2cTAP phenotype. In addition, the conserved fucosylation site is required for the dominant effect of the *WAK2cTAP*.

### Modification of WAKs

**Fig 2:**
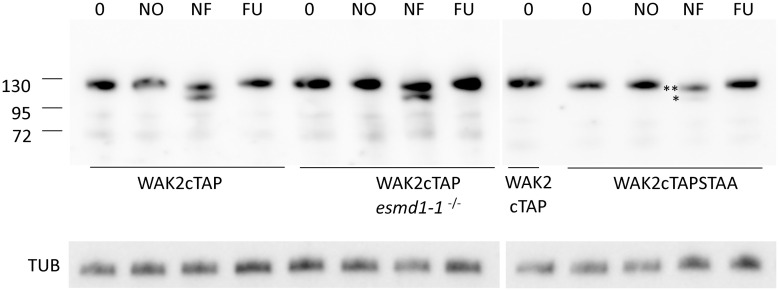
WAK2cTAP is modified by an N-linked sugar. Extracts of WAK2cTAP or WAK2cTAPSTAA (indicated below gels) were not treated (0) or treated with neuraminidase and O-glycosidase (NO), protein N-glycosidase F (NF) or fucosidase (FU) and run in a denaturing 5–20% polyacrylamide gel, and Western blotted to detect the TAP tag. Numbers on left indicate mw in KDa. The WAK2cTAP STAA samples were run in a separate gel from WAK2cTAP samples, and a WAK2cTAP marker was included for reference of migration. Below, the same samples were Western blotted with Tubulin antiserum as a loading control.

If indeed WAKs are a substrate for the ESMD1 O-fucosylation activity, a fucose should be detected covalently attached to the EGF domain. Attempts to analyze sufficient native or epitope tagged WAKs by immunoprecipitation and mass spec have failed to date. Moreover, native WAKs do not appear in proteomic whole cell analysis so that their modification state might be determined [36]. Therefore, the migration of WAK2cTAP and WAK2cTAP STAA before and after treatment with enzymes specific to glycosyl-protein modifications was used as a proxy for potential modification. A fucosidase, an O-glycosidase, and a N-glycosidase were incubated with extracts from plants expressing WAK2cTAP or WAK2cTAP STAA, run in a denaturing polyacrylamide gel, and Western blotted to detect the TAP tag, and the results are shown in Fig 2. WAK2cTAP migrates as a ca. 120KDa protein (lane 0 Fig 2) and neither O-glycosidase (NO) nor fucosidase (FU) cause a detectable shift in mobility. It is possible that the removal of a small O-linked sugar could not be detected by this gel analysis. However, the glycosidase specific to N-linked sugars, which can be of higher molecular weight than O- linked sugars, does cause a detectable molecular weight shift into two distinct bands (lane NF). This pattern is not affected if the extract is made from *esmd1-1*^-/-^ *WAK2cTAP* plants, indicating the shift is not dependent upon ESMD1, which is expected to create an O-linked specific modification. The WAK2cTAP STAA protein does migrate at a slightly lower molecular weight than WAK2cTAP (Fig 2), and is also not affected by fucosidase or O-glycosidase. However, the activity of the N-linked glycosidase is greatly hindered on WAK2cTAPSTAA as the lower band (*NF) is far fainter than for the treated WAK2cTAP. In addition, the upper band (**NF) is still slightly smaller than the untreated WAK2cTAP STAA indicating that there is some effect of the N-glycosidase on the STAA mutant. The results indicate that the STAA mutation causes a change in an N-linked glycosylation that is specific to the WAK protein. Since the largest mobility shift of WAK2cTAP seen by N-glycosidase treatment is not detected for WAK2cTAP STAA the modification is likely not on the attached TAP epitope.

### *esmd1* suppresses ROS and *FADlox* expression

**Fig 3:**
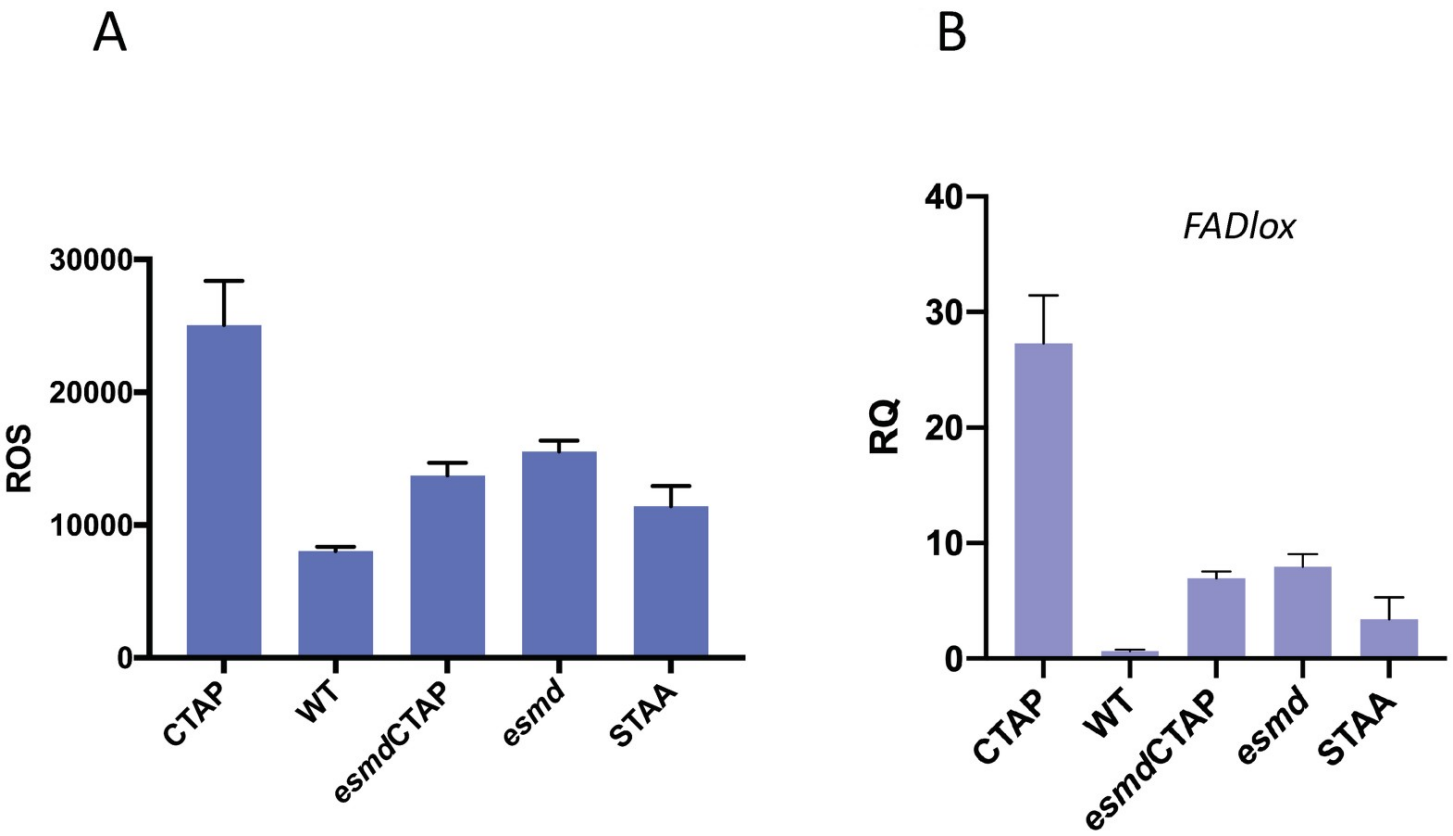
*esmd1-1* suppresses ROS accumulation and *FADlox* expression in *esmd1-1/WAK2cTAP* double mutant. A) ROS accumulation in total photon count/hr in indicated genotype (6 biological replicates). B) Relative gene expression (RQ) determined by RTqPCR for *FADLox*, relative to actin (3 biological replicates). An ANOVA indicated there was a difference between the samples (A; F(4,10) = 40.89 p<0.0001. B; F (4,10) = 73.16 p<0.0001). Tukey’s tests between each sample (S2 Fig) indicate pairwise differences (p<0.0001). Error bars indicate standard deviation.

One of the hallmarks of the WAK2cTAP phenotype is the constitutive induction of both ROS and *FADlox* gene expression [31]. To determine if *esmd1-1* also suppresses WAK2cTAP constitutive ROS, a luminol based assay using leaf discs [37] was used and the results are shown in Fig 3A. An ANOVA indicated there was a difference between the samples (F(4,10) = 40.89 p<0.0001). Tukey’s tests between each sample (S2 Fig) indicate that as expected and previously reported [31], ROS is elevated in WAK2cTAP relative to WT (p<0.0001). The STAA mutation reduces this constitutive ROS to levels that are still slightly higher than WT. *esmd1-1*^-/-^ had a slightly elevated ROS level relative to WT, and the double *esmd1-1*^-/-^ WAK2cTAP ROS level is also reduced relative to WAK2cTAP. RT-qPCR was used to assay the levels of *FADlox* expression, and Fig 3B shows that *WAK2cTAP* has elevated levels relative to WT (ANOVA F(4,10) = 73.16.p<0.001. Tukey’s tests shown in S2 Fig). The STAA mutation dramatically reduces the constitutive gene expression, as does the presence of *esmd1-1*. Thus *esmd1-1* suppresses the dwarf phenotype, and the constitutive ROS and *FADlox* expression of WAK2cTAP.

### *qua2-1* and *qua2-1*^-/-^*WAK2cTAP* mutant phenotypes

**Fig 4:**
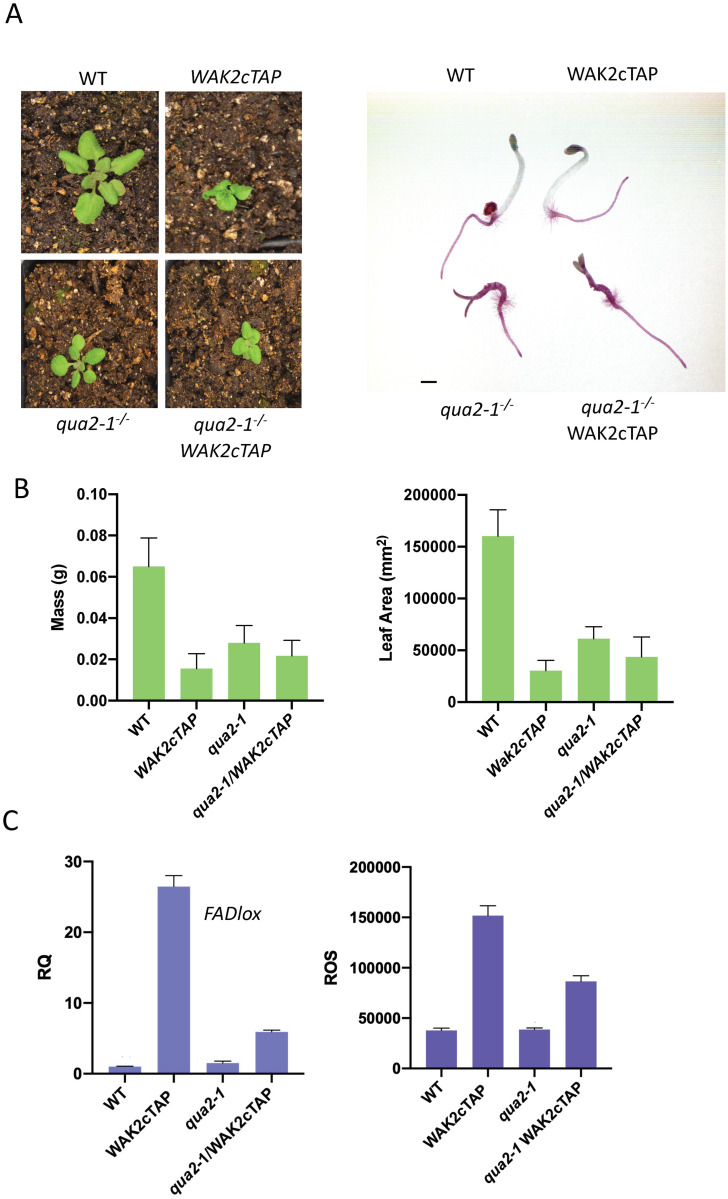
*qua2-1* and *WAK2cTAP* are likely in the same pathway. A) Left-Representative plants of indicated genotype grown under same conditions on soil. Right- Ruthenium Red dark grown hypocotyls of the indicated genotype. B) Quantification of wet mass and total leaf area of soil grown plants of indicated genotype (n = 6). Bar indicates 1 mm. C) Left-ROS accumulation in total photon count/hr in indicated genotype (6 biological replicates). Right-Relative gene expression (RQ) determined by RT-qPCR for *FADLox*, relative to actin (3 biological replicates). ANOVA and Tukey’s tests are reported in the text and S2 Fig. Error bars indicate standard deviation.

WAKs were proposed to be involved in an ESMD1 dependent signaling pathway that regulates pectin-based adhesion [22]. Since *esmd1-1* suppresses the adhesion defective *qua2-1*, a mutation of a pectin methyltransferase, and is required for proper cell adhesion [22], it was of interest to determine if and how the WAK2cTAP phenotype would be impacted by the *qua2-1* mutant. If *qua2-1* and *wak* mutants affect different pathways, they would be expected to be additive, but if they are in the same pathway, then the double mutant phenotype would be expected to be similar to WAK2cTAP. Homozygous *qua2-1* mutants were crossed to the homozygous *WAK2cTAP* line, and the resulting F_2_ progeny were screened by Western blot for WAK2cTAP expression and sequencing of *QUA2* PCR products for plants homozygous for *WAK2cTAP* and *qua2-1* alleles. Phenotypes of the single *qua2-1* mutant and the *qua2-1*^-/-^*WAK2cTAP* double mutant were compared to WT and WAK2cTAP plants visually as well as by wet weight and leaf area to determine if there was any interaction between the alleles (Fig 4). As expected, due to the reduced pectin content in the cell wall, *qua2-1* mutants displayed a dwarfed phenotype in comparison to WT, but were nevertheless larger than WAK2cTAP (Fig 4A). *qua2-1*^-/-^*WAK2cTAP* appeared larger than WAK2cTAP but smaller than *qua2-1*^-/-^. Notably, the twisted and curled leaf shape of WAK2cTAP were not present in either *qua2-1* nor *qua2-1*^-/-^*WAK2cTAP*. Dark grown hypocotyls were also stained with Ruthenium Red which binds to pectin and stains wild type roots but cannot penetrate the wall to stain wild type hypocotyls, and has been used to detect adhesion defects and pectin changes in hypocotyls [22]. Fig 4A also shows that the WAK2cTAP has no effect on the red staining detached cell phenotype of *qua2-1*. An ANOVA of wet weight and leaf area indicated there was a difference between the samples (Area;F(3,18) = 56.24 p<0.0001. Mass; F (3,22) = 36.56 p<0.001). Tukey’s tests between each sample (S2 Fig) indicated that there is a difference between the mass and leaf area of *qua2-1*^-/-^ and WT and a difference in leaf area between *qua2-1*^-/-^ and WAK2cTAP plants (p<0.0001, Fig 4B). In contrast to *qua2-1*, the *qua2-1*^-/-^*WAK2cTAP* double mutant does not demonstrate a difference in size by either mass or leaf area to WAK2cTAP (Fig 4B). The lack of difference in mass and measured size between the WAK2cTAP and *qua2-1*^-/-^*WAK2cTAP* suggests that the hyperactive WAK2cTAP phenotype is not additive with mutations in the QUA2 pectin methyltransferase. However, the double mutant no longer has curled leaves and the results also suggest that *qua2-1* and WAK2cTAP impact common pathways, and this is what one would expect for an allele of a biosynthetic enzyme and a receptor bound to the product of that enzyme. The loss of leaf curling in the double mutant might be linked to a loss of cell adhesion that could suppress the twisting phenotypes, most likely preventing supracellular mechanical coupling of adjacent cells [41]. The levels of ROS and *FADlox* expression in the double mutant were also measured, and the results are shown in Fig 4C. An ANOVA indicated there was a difference between the samples (F(3,20) = 510.9 p<0.0001). Tukey’s tests between each sample (S2 Fig) indicates, as expected, WAK2cTAP shows higher levels of ROS and *FADlox* expression relative to WT and *qua2-1*, but these are abated but not completely reduced in the *qua2-1*^-/-^*WAK2cTAP* plant, consistent with the leaf phenotypes. It was reported that *qua2-1* seedlings grown *in vitro* had elevated levels of *FADlox* expression and showed a strong phenotype relative to WT [22] but neither the *FADlox* change nor strong phenotype are detected in the soil grown leaves measured here.

### *esmd1* suppression of *qua2-1* does not require WAKs

**Fig 5:**
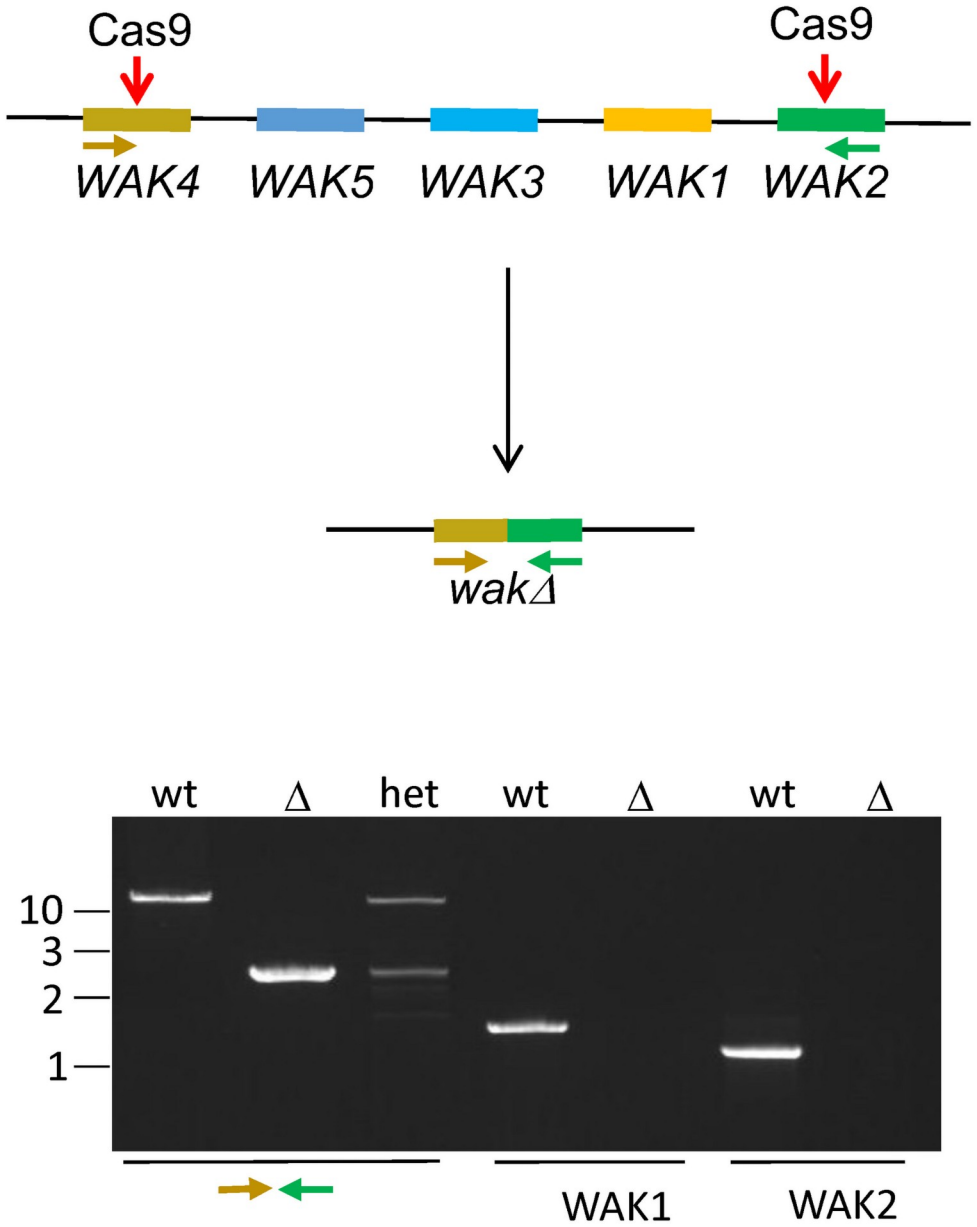
A 25 Kb WAK locus deletion. Cartoon depicts the arrangement of 5 *WAKs* in the Arabidopsis genome. Red arrows indicate site of Cas9 cleavage. Lower cartoon shows the result of the deletion fusing *WAK4* with *WAK2*. Lower gel shows ethidium bromide stained PCR products using *WAK4* forward and *WAK2* reverse primers (brown and green arrows) on genomic DNA isolated from the indicated genotype above gel. *WAK1* and *WAK2* were amplified using gene specific primers [42]. Numbers on left indicate Kb. Arrows indicate location of primers used for PCR.

WAKs were predicted to be a component of the ESMD1 signaling mechanism that suppresses a loss of cell adhesion due to pectin deficiency in *qua*2-1^-/-^. A robust test of this prediction would be to determine if WAKs are required for the suppression. However, to date only single loss of function *WAK* alleles have been available and these have no or weak phenotype and it is assumed that there is functional redundancy within the tightly clustered family of five genes [40]. Therefore, CRISPR was used to induce a deletion of the 25 kb locus that contains the 5 *WAK* genes (termed *wakΔ*^-/-^). This mutant *wak*Δ^/-^ was first characterized, and then combined into a triple mutant of *esmd1*^-/-^ *qua2*^-/-^*wakΔ*^-/-^. Sg RNAs were designed to target the 5’ most *WAK4* gene and the 3’-most *WAK2* gene of the *WAK* locus (Fig 5 and S3A Fig), and first generation transformed wild type Arabidopsis (T1) were selected for expression of the sg RNAs by the linked hygromycin resistance, Cas9, and seed expressed RFP. DNA from twelve T1 were tested for the presence of a deletion using PCR primers in the *WAK4* and *WAK2* genes as shown in Fig 5. Conditions were established to detect the 25 kb wild type PCR product, and a successful deletion 2.8 kb PCR product. Twelve plants contained both PCR products, and one of these was self-crossed. The resulting T2 plants were then screened for the loss of an RFP marker linked to Cas9 and the two sg RNA genes, and 52 of these were planted on soil. These RFP- plants were then screened by PCR for the 2.8 KB deletion band with the assumption that since Cas9 was lost, the deletion was necessarily inherited. Three individuals were heterozygous for the WT 25 Kb wild type locus and the 2.8 kb deletion locus PCR bands, and one of these T2 plants was then self-crossed, and a T3 individual homozygous for the deletion was isolated and termed *wakΔ*^-/-^_._ This plant had no *Cas9* gene as determined by PCR. No PCR product for *WAK1* and *2* could be detected in the homozygous mutant (Fig 5). The deletion 2.8 Kb PCR band was sequenced and the expected deletion was indeed observed to join the two sg cleavage sites together, leaving the 5’-most coding region for the extracellular domain of WAK4 and the last half of the kinase domain of WAK2 (S3A Fig). The predicted WAK4-2 protein contains 340 amino acids of the WAK4 ECM domain fused to an out of frame coding region from the kinase domain of WAK2, and there is no transmembrane domain left. The WAK4 WT ECM domain is 356 amino acids. Thus the WAK4 kinase domain, WAK 3,5, 1 and the 5’ coding region of WAK2 extracellular domain are deleted. Five other attempts using different sg RNAs to remove the 5’ end of WAK4 have not been successful despite 4 years and 1000’s of plants screened.

Surprisingly the *wak*Δ^-/-^ individual had no obvious phenotype on soil. However, when grown on agar the roots were shorter, as observed for plants having only the *wak2*^-/-^ mutation (t test, p< 0.05, S3B Fig) [28, 43]. RNA seq analysis of soil grown leaves comparing WT and *wak*Δ^-/-^ indicated that there was no RNA expression from *WAK1*,*3*, *and 5* (S5 Fig). RNA was detected from the 5’ end of *WAK4* that had not been deleted, but the padj is greater than 0.05 and is therefore not significant. In addition, *WAK4* is normally expressed only at very low levels in hypocotyls and leaves (readcount column S4 Fig). *WAK2* was also expressed in *wakΔ*^-/-^ at 8 logs less than WT (S4 Fig). The expression of *WAK4* and *2* represent only the fragments of the genes that remained as determined using Novogen supplied Bam files and the IGV program from the Broad Institute (see Materials and methods). Consistent with a lack of visible phenotype of *wak*Δ^-/-^, RNA seq analysis shows that only several genes were changed in their expression relative to wild type, and these included a glycosyltransferase (At1g05675, UGT784E1) and *LURP1* that were several fold upregulated (log 2, S4 Fig). *LURP1* responds to infection by *Hyaloperonospora parasitica* [44]. T-DNA mutations in these two genes were separately combined with *wak*Δ^-/-^ but the double mutants also appeared WT suggesting that the upregulation might not compensate for effects of the *wak*Δ^-/-^. Exploratory tests of infection with powdery mildew and separately with Botrytis of *wak*Δ^-/-^ and WT plants showed no difference in infection, and therefore further analysis was not perused. More extensive analysis of pathogen sensitivity will require the screening of numerous pathogens. Two other genes, At3g30720 (protein QQS) and At5g65080 (MADS- box transcription factor), were also upregulated 2.2 and 3.7 log_2_ fold, respectively (S4 Fig). Protein QQS is annotated in TAIR as being involved in regulating carbon and nitrogen allocation to starch and protein, and the MADS-box transcription factor has undefined gene targets, but neither of these were investigated further.

**Fig 6:**
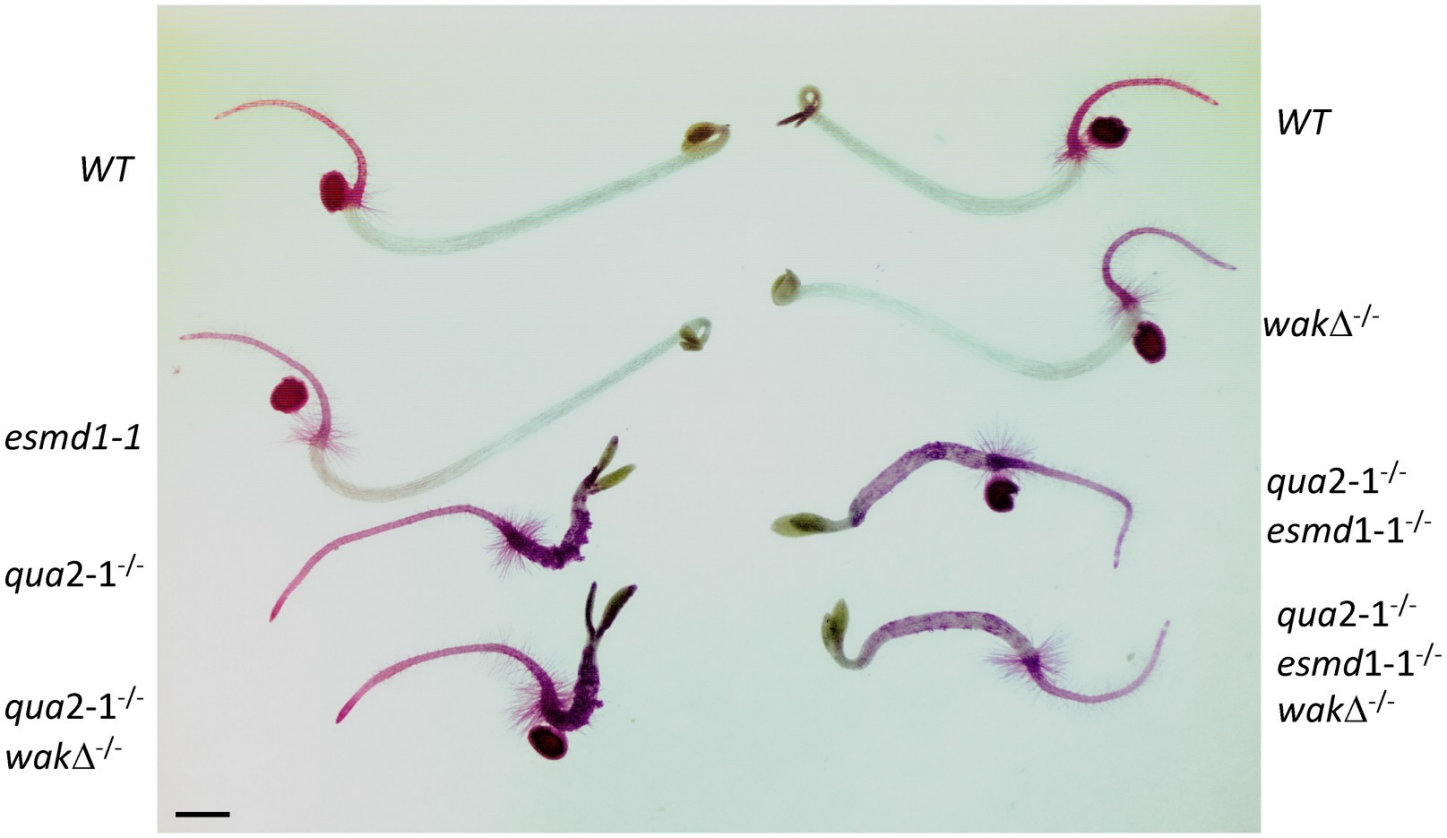
*esmd1* does not require WAKs for suppression of *qua2-1*. Representative dark grown, Ruthenium Red stained hypocotyls of the indicated genotype. Bar indicates 1 mm.

The wakΔ^-/-^ was then crossed to an *esmd1-1*^-/-^*qua2-1* ^-/-^ individual, and a *wak*Δ^-/-^ *esmd1-1*^-/-^ *qua2-1* ^-/-^ triple mutant was identified in the F2 population by PCR of the loci and sequencing. Fig 6 shows the progeny of this plant grown in the dark for 4 days and stained with Ruthenium Red to detect pectin and adhesion defects, as compared to single mutants and wild type. While *qua2-1* ^-/-^ displays red hypocotyl staining and adhesion defects as expected [19], *esmd1-1* can partially suppress this phenotype (*esmd1-1*^-/-^*qua2-1* ^-/-^). However, the addition of wakΔ^-/-^ has no effect on the ability of *esmd1-1* to suppress *qua2-1*, indicating that the WAK locus is not required for ESMD1 suppression. The results also indicate that the *qua2-1* ^-/-^ phenotype is not dependent upon WAKs.

### Effects on ROS

**Fig 7:**
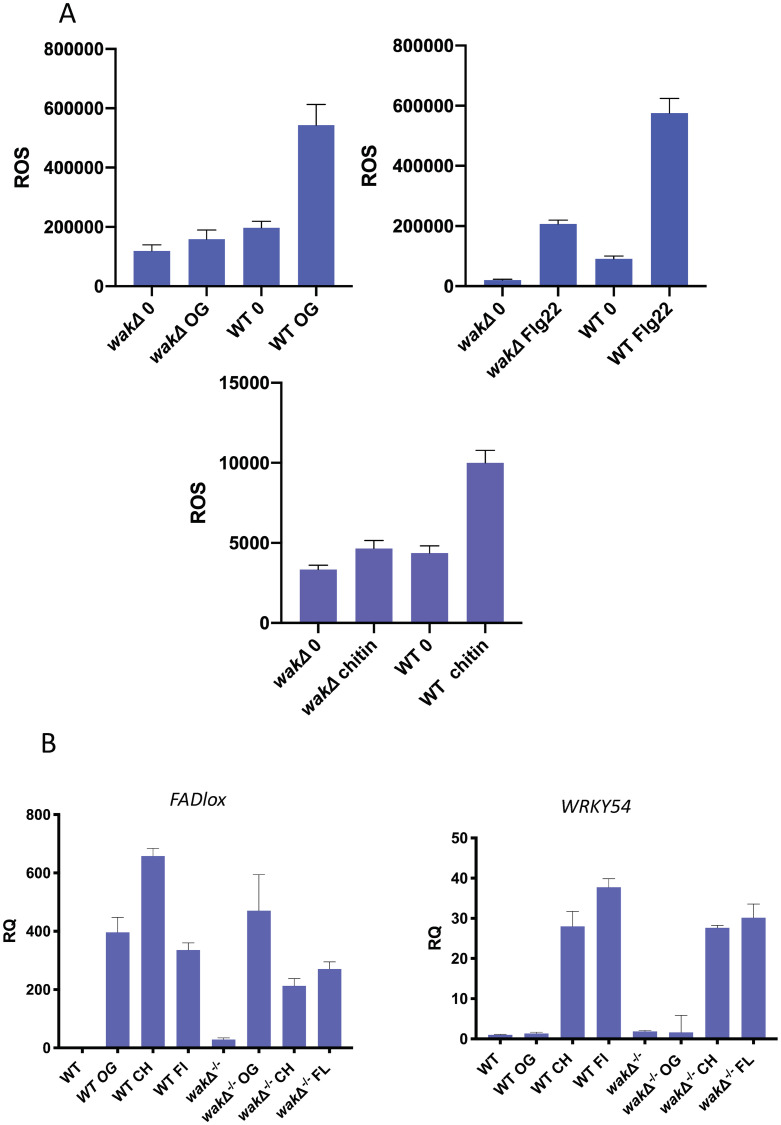
WAKs are required for OG, chitin and Flg22 activation of ROS but not transcription. A) left column, ROS accumulation in total photon count/hr in indicated genotype (6 biological replicates). B) right column, Relative gene expression (RQ) determined by RT-qPCR for *FADLox or WRKY54*, relative to actin for the indicated genotype and inducer (3 biological replicates). CH; chitin, FL; Flg22. ANOVA and Tukey’s tests are reported in the text and S2 Fig. Error bars indicate standard deviation.

While *wak*Δ^-/-^ has no dramatic phenotype on soil, and is not required for *esmd1-1* suppression of adhesion defects, it was of interest to determine if the mutation affected the perception of pectin fragments, as some evidence suggests that WAKs can be activated by OGs [32, 40]. In addition, a previous analysis of oligogalacturonic acid (OG) induction of protein phosphorylation indicated that LIK1 was induced to be phosphorylated [36], and since LIK1 associates with the chitin receptor CERK1 [45], chitin signaling was implicated. For comparison, bacterial flagellin (Flg22) induced signaling was also included in the analysis. All three stimuli, OG (dp 9–15), chitin and Flg22 activate an extracellular ROS response [46, 47], although chitin appear to elicit a lower response. The ROS accumulation after the exposure of leaf discs to OGs, chitin and Flg22 was measured over 60 minutes and the results are shown in Fig 7A. An ANOVA indicated that there was a difference between the samples (OG; F(3,20) = 134.6 p<0.0001. Chitin F(3,8) = 94.49 p<0.0001. Flg22; F(3,8) = 277.3 p<0.0001). Tukey’s tests between each sample (S2 Fig) indicated that while WT leaves showed a dramatic increase for each type of treatment, *wakΔ*^-/-^ exhibited a significant loss of response in each stimulation. WAK2cTAP has an elevated constitutive ROS (Fig 3A), and OG treatment does not further increase this [30]. However, in the *emsd1* suppressed WAK2cTAP the levels are similar to WT, but still inducible by OGs (S5 Fig, Tukey’s p<0.0001 S2 Fig).

The ROS activation is also accompanied by the activation of numerous genes where *FADlox* has been used as a reporter for OGs, while *WRKY54* is activated by Flg22 and chitin [31, 48]. The levels of induction were measured by RT-qPCR for each of the three treatments of seedlings, and the results are shown in Fig 7B. An ANOVA of indicated there was a difference between the samples (FADlox; F(7,16) = 56.68) p<0.0001.WRKY53 F(7,14) = 102.2 p<0.0001). Tukey’s tests between each sample (S2 Fig) indicated that unlike the abatement of ROS activation, *wak*Δ^-/-^ had a *FADlox* induction similar to WT indicating that WAKs are not required for the OG transcriptional response. While there was a reduction in the chitin and Flg22 response relative to wild type, there was still a significant response relative to no inducer. The *wak*Δ^-/-^ appeared to have no effect on the *WRKY54* induction by chitin or Flg22.

## Discussion

The cell wall is comprised of an array of cellulose, hemicellulose, pectin, and proteins and forms a boundary and adhesion surface between cells that regulates development and impact the health of the plant. Until recently, cell adhesion was thought to be a direct linked to pectin content within the cell wall. However, the ability of the cell wall to compensate for reduced pectin content and still demonstrate proper adhesion points to the existence of an undefined signaling mechanism [22]. As WAKs are receptor kinases and have the ability to bind to both wall pectin and short pectin fragments known as oligogalacturonides (OGs), WAKs serve as a prime candidate for this pectin signaling cascade [40]. Using several alleles of the dominant *WAK2cTAP* and the mutants *esmd1-1* and *qua2-1*, which likely encode a pectin O-fucosyltransferase and methyltransferase respectively [19, 20, 22], double mutants were constructed to identify possible genetic interactions. The results show that *esmd1-1* partially suppresses the dwarf WAK2cTAP phenotype as well as constitutive ROS production, a hallmark of the stress response. Further, results suggest that the potential WAK O-fucosylation site is required for the WAK2cTAP phenotype since the serine/threonine to alanine mutation at the potential fucosylation site abates the stress phenotype. Since ESMD1 is a putative fucosyl transferase, it is possible that a modification of WAK in the EGF-like repeats plays a role in WAK activity. However, the WAKs appear not to be required for the ability of *esmd1-1* to suppress the pectin deficient *qua2-1* adhesion mutant that causes a reduction in wall pectin content, as the *esmd1*^-/-^ *qua2*^-/-^*wak*Δ^-/-^ mutant appears to be like the *esmd1*^-/-^ *qua2*^-/-^.

Since the STAA mutation reduced the effects of WAKcTAP, and *esmd1* suppresses WAKcTAP it was expected that an O-fucosylation would be detected. However, a shift in mobility of the WAKcTAP could not be detected after fucosylase treatment, nor was there a difference in an *esmd*^-/-^ background. It is quite possible that the gel mobility shift is too small to be detected and the modification will need to be explored using mass spectrometry once WAKs can be identified in extracts. This awaits the development of a method to isolate native WAKs or to detect them using mass spec analysis in whole cell or fractionated extracts which to date has proven difficult. It remains possible that ESMD1-mediated O-fucosylation is growth condition dependent, and the experiments here have not discovered the particular conditions. An N-linked glycosylation is detected, perhaps because N-Linked modifications are often of higher molecular weight than O-linked sugars, but the role and location of the N-linked modification in WAK is not yet defined. This N-linked modification, however, is influenced by the presence of the ST in the potential fucosylation site, but the cause of this is not known. The results suggest that while ESMD1 may indeed use WAKs as substrate, the ESMD1 related signaling pathway that regulates pectin adhesion without increasing pectin content engages yet another type of signaling pathway distinct from WAK. There are six other proteins in the Arabidopsis genome that encode proteins with a potential O-fucosylation site, and these all are predicted to encode a G-type lectin S-receptor-like serine/threonine-protein kinase [22]. It will be of interest to determine if this family of receptors is required for the suppression of *qua2-1* by *esmd1-1*, yet there is no evidence that these type of receptors are involved in the cell wall. One additional potential ESMD1 substrate may well be ExAD, a cell wall specific glycoprotein glycosyl transferase that contains a fucosylation consensus sequence [49]. ExAD likely modifies Extensins which in turn can affect the integrity of the cell wall, and so an exploration of ESMD1 and ExAD interactions will be important for understanding cell wall signaling. The receptor kinase FERONIA is also thought to bind to pectin [50], but there is no evidence that it is either fucosylated or involved in a WAK or ESMD1 related pathway. The nature of the ESMD1 enzymatic activity also needs to be characterized, as while it has sequence similarity to metazoan counterparts, there may be substrate specificity differences [51]. It is also possible that the suppression of *qua2-1* by *esmd1-1* is not directly through a pectin related pathway but more involved in reducing a stress related event that somehow restores adhesion, and this would explain why *esmd1-1* suppresses WAK2cTAP. Indeed, a recent report shows that a reduction in peroxidase activity can partially suppress *qua2-1* [52]. It may well be that *esmd1-1* can suppress multiple different types of cellular stresses that contribute to the integrity of the cell wall.

Additionally surprising was the absence of a dramatic growth phenotype for *wakΔ*^-/-^ individuals as previous work has suggested that the WAKs play redundant roles in cell expansion and growth. It remains possible that the remaining WAK4 extracellular domain in the *wakΔ*^-/-^ deletion may provide some residual function. The predicted protein contains 340 of the 356 amino acids of the WAK4 ECM domain fused to an out of frame coding region from the kinase domain of WAK2, and there is no transmembrane domain left. Thus if this WAK4-2 fusion protein were expressed then it would be secreted. Attempts to create a larger *WAK* deletion have not been successful but are needed to determine if this portion of WAK4 is sufficient to provide WAK function. Given the *WAK4-2* fusion RNA expression level is very low relative to *WAK1* and 2, and that it would be expected to be secreted and not membrane bound, it is unlikely to be the cause of the lack of visible phenotype.

The *wakΔ*^-/-^ growth phenotype is similar to that of a single *wak2* ^-/-^ and we are left with the possibility that there remains further functional redundancy. The Arabidopsis genome also encodes 26 other proteins that contain both the EGF-like repeats and kinase domains that are characteristic of WAKs [53]. These genes, known as *WAK-like (WAKL)*, are present in other species and mutant alleles have been shown to convey pathogen and disease resistance, often as a gain of function allele [54–57]. Though there is great divergence between WAKs and WAKLs and the WAKLs are not known to associate with pectin or the cell wall, the potential for coordinated signaling between the WAKs and WAKLs should not be ignored [40]. However, none of the WAKLs contain potential conserved fucosylation sites and thus are not candidates for ESMD1 substrates.

While WAKs may not be required for *esmd1* suppression of pectin deficiency, surprisingly the *wak*Δ^-/-^ mutation causes a dramatic reduction of the ROS response to a variety of elicitors, including OGs, bacterial flagellins, and chitin. While it is clear that WAKs bind to pectin polymers *in vivo* and *in vitro* [33, 40, 43] evidence that they serve as the receptor specifically for OGs with lengths (dp) 9–15 is somewhat weaker [32]. Indeed, phosphoproteomics of OG induced plants failed to identify a dedicated phosphorylation pathway, but rather modifications in proteins that regulate endocytosis [36]. The results here show that WAKs are required for the ROS response to OGs dp 9–15, but this does not demonstrate that they are an OG receptor, even though WAKs bind to pectins. OG dp 9–15 have previously been presumed to mimic a true pathogen infection, yet this interpretation is complicated by the recent observation that shorter OGs and not ones of dp 9–15 are found upon pathogen infection [58]. This later report did find that the shorter OGs generated by pathogens can induce a plant response, yet these are not known to activate or bind WAKs with high affinity [33, 40]. Indeed, the results reported here also show that WAKs are not required for the *FADLox* transcriptional response that is the hallmark of a dp 9–15 OG activation [48]. The most parsimonious conclusion is that WAKs help to mediate a ROS response to multiple elicitors, yet are not essential for the activation of OGs, Flg22, or chitin specific signaling that leads to transcriptional response specific to each inducer. It may be that the ROS response itself leads to some level of *FADlox* expression that is observed. This is in agreement with the suggestion that cereal WAKs may be involved in a basal resistance to pathogens [57]. There may well therefore be additional receptors required for the sensing of OGs if there is indeed a specific pathway, and this may be related to the reason that *esmd1* suppression of *qua2-1* is not affected by the loss of WAKs. Since the dominant allele WAK2cTAP does induce a response that mimics a pathogen response, it is possible that WAKs are accessory receptors to multiple pathways that are specific to different biotic agents [40].

In tomato, SIWAK1 expression appears to be induced by pathogen induced ROS which in turn leads to its association with FLS2 and activation of late transcriptional responses [59]. The difference in the role of the Arabidopsis WAKs and SIWAK1 may be due to the observation that the WAKs are a large family having EGF containing extracellular domains and highly similar kinases, and possibly divergent function. Indeed, in Arabidopsis there are 5 WAKs but 26 other WAK-like (WAKL) proteins [53] and the latter are not known to be wall associated. Only the Arabidopsis WAK1-5 are known to bind to pectin and are cross linked to the cell wall so that, unlike the WAKs of crop plants, they do not appear in a partially soluble fraction in association with other receptors [40]. It remains quite possible that many of the WAK-like genes reported in other species [60, 61], including tomato S1WAK1, encode WAKLs and not Wall associated kinases (WAK1-5). An analysis of the WAK -like ((WAKLs) receptors in Arabidopsis will address this suggestion. It is also possible that WAKs play a slightly different role in Arabidopsis and tomato.

The WAKs and /or WAKLs appear to play important roles in the resistance to a variety of pathogens in many species, especially in crop plants where the family has greatly expanded in size [57]. Since the fucosylation site appears to abate the effect of WAK2cTAP which mimics a pathogen response through a constitutive hyperactivation, it follows that the fucosylation site might play a role in the pathogen response. However, exploratory tests of Botrytis and Powdery Mildew infection of wakΔ^-/-^ plants showed no change in response relative to WT and since the WAKLs do not contain the fucosylation site, it argues against such a role. An understanding of the WAK and WAKL family awaits the creation of a plant that lacks all of these genes and a systematic testing of a variety of pathogens and their effect on the *wak/wakl* mutants.

## Supporting information

S1 Fig Shown is a cartoon of the domains structure of WAKs, and below the sequence of the EGF repeats EGF1 and EGF2. Red indicated the conserved cysteines, and green the conserved Serine and threonines that form the consensus fucosylation site. Subscripts below the Cs indicate the position within one EGF repeat. TM; transmembrane domain.
(PDF)

S2 Fig ANOVA and Tukey’s tests for data presented in the manuscript. (PDF)

S3 Fig A. Top is shown the consensus SgRNA, and the annealed pairs selected for WAK4 in green, and WAK2 in mustard. Red bases indicate the required NGG. The result of the Sg cut and fused WAK4-2 sequence is shown at the bottom. B. Root length in mm of WT and *wak*Δ seedlings grown on MS agar. C. List of oligonucleotides used in the analysis.
(PDF)

S4 Fig RNA seq results of up and down changes in expression between WT and *wak*Δ. No other significant changes (padj<0.05) in the transcripts were detected. Red highlights the *WAK* genes, and green other genes.
(PDF)

S5 Fig ROS accumulation in total photon count/hr in indicated genotype (6 biological replicates). ANOVA and Tukey’s tests are reported in the text and S2 Fig.
(PDF)

S6 Fig Raw image file for Western blots shown in Figs 1 and 2. (PDF)
