# Supplementary material for: Effects of Arabidopsis wall associated kinase mutations on ESMERALDA1 and elicitor induced ROS
Source: PLoS One. 2021 May 20;16(5):e0251922. doi: 10.1371/journal.pone.0251922 (PMC8136723; doi:10.1371/journal.pone.0251922)
Supplement: S1 Fig — Red indicated the conserved cysteines, and green the conserved Serine and threonines that form the consensus fucosylation site. Subscripts below the Cs indicate the position within one EGF repeat. TM; transmembrane domain. (PDF) [file pone.0251922.s001.pdf]

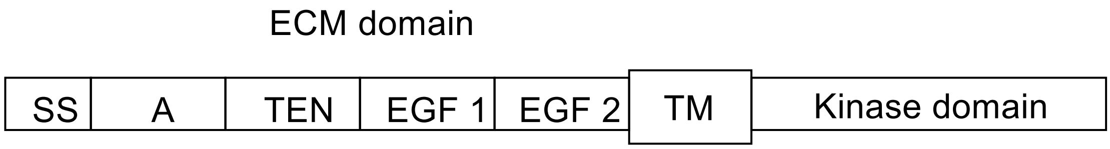

	C_1 6-10 C_2 3 ST C_3 7-10 C_4 1 C_5 11-13 C_6 C_2 4-5 S/T C_3
WAK1 EGF1	DWSVGNQTCEQVGSTSI . . CGGNSTCLDSTPRNGYI CRCNE GFDGNP YLSAGC
WAK1 EGF2	QDVNECTTSSTIHRHNCSDPKT CRNKVGGFY.. CKCQS GYRL D. TTTMSC
WAK2 EGF1	DWSIGDKT CKQVEYRGV.. CGGNSTCFDSTGGTGYNCKCLE GFEGN PYLPN GC
WAK2 EGF2	QDINEC . . . . . ISSRHNCSEH STCENTKGSFN . . CNCPSG YRK DSLNS . .C
WAK3 EGF1	DWSIGNQT CEQAGSTRI . .CGKNSSCYNSTT R N GYICKCNEGYDGNPYRSE GC
WAK3 EGF2	KDIDECISDTH . . . . .NCSDPKTCRNRDGGF. . DCKCPSGYDLNSS . . MSC
WAK4 EGF1	DWSIRGETCGQVGEKK. ..CGVNGICSN SASGIGY TCKCKGGFQGNPYLQNGC
WAK4 EGF2	QDINECTTANPIHKHNCSGDSTCENKLGHFR . . CNCRSRYELNTTT . NTC
WAK5 EGF1	DWSIGNQTCEQVVGRNI . .CGGNSTCFDSTRGKGY NCKCLQGFDGNPY LSDGC
WAK5 EGF2	QDINECTTRIHN . . . . . CSD TSTCENTLGSF . . . HCQCPSGSDLNTTT...MSC
