# Supplementary material for: Effects of Arabidopsis wall associated kinase mutations on ESMERALDA1 and elicitor induced ROS
Source: PLoS One. 2021 May 20;16(5):e0251922. doi: 10.1371/journal.pone.0251922 (PMC8136723; doi:10.1371/journal.pone.0251922)
Supplement: S2 Fig — (PDF) [file pone.0251922.s002.pdf]

ANNOVA and Tukey's for Kohorn et al Figs.

Fig 1B Area

F (5, 28) = 18.87	P<0.0001				
Tukey's multiple comparisons test	Mean Diff.	95.00% CI of diff.	Significant?	Summary	Adjusted P Value
WT vs. Wak2cTAP	129358	79677 to 179040	Yes	****	<0.0001
WT vs. esmd	-5782	-55464 to 43900	No	ns	0.9992
WT vs. esmd/WAK2cTAP	80111	32741 to 127480	Yes	***	0.0002
WT vs. Wak2cTAP STAA	44833	-2537 to 92203	No	ns	0.0716
WT vs. esmd/WAK2cTAP STAA	57781	10412 to 105151	Yes	*	0.0101
Wak2cTAP vs. esmd	-135140	-187031 to -83250	Yes	****	<0.0001
Wak2cTAP vs. esmd/WAK2cTAP	-49248	-98930 to 433.8	No	ns	0.0531
Wak2cTAP vs. Wak2cTAP STAA	-84525	-134207 to -34844	Yes	***	0.0002
Wak2cTAP vs. esmd/WAK2cTAP STAA	-71577	-121259 to -21896	Yes	**	0.0018
esmd vs. esmd/WAK2cTAP	85893	36211 to 135574	Yes	***	0.0002
esmd vs. Wak2cTAP STAA	50615	933.3 to 100297	Yes	*	0.0439
esmd vs. esmd/WAK2cTAP STAA	63563	13881 to 113245	Yes	**	0.0064
esmd/WAK2cTAP vs. Wak2cTAP STAA	-35278	-82647 to 12092	No	ns	0.2372
esmd/WAK2cTAP vs. esmd/WAK2cTAP STAA	-22329	-69699 to 25040	No	ns	0.7029
Wak2cTAP STAA vs. esmd/WAK2cTAP STAA	12948	-34421 to 60318	No	ns	0.9582

Fig 1B Mass

F (5, 34) = 14.63	P<0.0001				
Tukey's multiple comparisons test	Mean Diff.	95.00% CI of diff.	Significant?	Summary	Adjusted P Value
WT vs. WAK2cTAP	0.04944	0.02699 to 0.07190	Yes	****	<0.0001
WT vs. esmd	0.005	-0.01960 to 0.02960	No	ns	0.9893
WT vs. esmd/WAK2cTAP	0.029	0.003200 to 0.05480	Yes	*	0.0201
WT vs. STAA	0.005	-0.01801 to 0.02801	No	ns	0.9855
WT vs. esmd STAA	0.006667	-0.01793 to 0.03127	No	ns	0.9622
WAK2cTAP vs. esmd	-0.04444	-0.06690 to -0.02199	Yes	****	<0.0001
WAK2cTAP vs. esmd/WAK2cTAP	-0.02044	-0.04421 to 0.00332	No	ns	0.1258
WAK2cTAP vs. STAA	-0.04444	-0.06515 to -0.0237	Yes	****	<0.0001
WAK2cTAP vs. esmd STAA	-0.04278	-0.06523 to -0.02033	Yes	****	<0.0001
esmd vs. esmd/WAK2cTAP	0.024	-0.001800 to 0.04988	No	ns	0.0805
esmd vs. STAA	0	-0.02301 to 0.02301	No	ns	>0.9999
esmd vs. esmd STAA	0.001667	-0.02293 to 0.02627	No	ns	>0.9999
esmd/WAK2cTAP vs. STAA	-0.024	-0.04829 to 0.00029	No	ns	0.0544
esmd/WAK2cTAP vs. esmd STAA	-0.02233	-0.04813 to 0.00346	No	ns	0.1217
STAA vs. esmd STAA	0.001667	-0.02134 to 0.02468	No	ns	>0.9999

Fig 3A

F (4, 10) = 40.89	P<0.0001				
Tukey's multiple comparisons test	Mean Diff.	95.00% CI of diff.	Significant?	Summary	Adjusted P Value
CTAP vs. WT	17039	12372 to 21706	Yes	****	<0.0001
CTAP vs. *esmd*CTAP	11333	6666 to 16000	Yes	****	<0.0001
CTAP vs. esmd	9538	4871 to 14205	Yes	***	0.0004
CTAP vs. STAA	13662	8995 to 18329	Yes	****	<0.0001
WT vs. *esmd*CTAP	-5706	-10373 to -1039	Yes	*	0.0161
WT vs. esmd	-7501	-12168 to -2834	Yes	**	0.0025
WT vs. STAA	-3377	-8044 to 1290	No	ns	0.1975
*esmd*CTAP vs. esmd	-1795	-6462 to 2872	No	ns	0.7161
*esmd*CTAP vs. STAA	2329	-2338 to 6996	No	ns	0.5057
esmd vs. STAA	4124	-542.8 to 8791	No	ns	0.0903

Fig 3B

F (4, 10) = 73.16	P<0.0001				
Tukey's multiple comparisons test	Mean Diff.	95.00% CI of diff.	Significant?	Summary	Adjusted P Value
CTAP vs. esmd	19.35	13.64 to 25.06	Yes	****	<0.0001
CTAP vs. esmdCTAP	20.34	14.63 to 26.05	Yes	****	<0.0001
CTAP vs. STAA	23.86	18.15 to 29.58	Yes	****	<0.0001
CTAP vs. WT	26.68	20.97 to 32.40	Yes	****	<0.0001
esmd vs. esmdCTAP	0.9898	-4.724 to 6.704	No	ns	0.9766
esmd vs. STAA	4.512	-1.202 to 10.23	No	ns	0.1439
esmd vs. WT	7.335	1.621 to 13.05	Yes	*	0.0119
esmdCTAP vs. STAA	3.522	-2.192 to 9.236	No	ns	0.3196
esmdCTAP vs. WT	6.345	0.6308 to 12.06	Yes	*	0.0285
STAA vs. WT	2.823	-2.891 to 8.537	No	ns	0.5148

continued

Fig 4A Area

F (3, 18) = 56.24 P<0.0001

Tukey's multiple comparisons test	Mean Diff.	95.00% CI of diff.	Significant?	Summary	Adjusted P Value
WT vs. Wak2cTAP	129358	97071 to 161645	Yes	****	<0.0001
WT vs. qua2-1	98993	66706 to 131280	Yes	****	<0.0001
WT vs. qua2-1/WAk2cTAP	116430	85646 to 147215	Yes	****	<0.0001
Wak2cTAP vs. qua2-1	-30365	-64088 to 3357	No	ns	0.0863
Wak2cTAP vs. qua2-1/WAk2cTAP	-12928	-45215 to 19359	No	ns	0.6754
qua2-1 vs. qua2-1/WAk2cTAP	17437	-14850 to 49724	No	ns	0.4432

Fig4B Mass

F (3, 22) = 36.56 P<0.0001

Tukey's multiple comparisons test	Mean Diff.	95.00% CI of diff.	Significant?	Summary	Adjusted P Value
WT vs. WAK2cTAP	0.04944	0.03572 to 0.06317	Yes	****	<0.0001
WT vs. qua2-1	0.037	0.02123 to 0.05277	Yes	****	<0.0001
WT vs. qua2-1/WAK2cTAP	0.04333	0.02830 to 0.05837	Yes	****	<0.0001
WAK2cTAP vs. qua2-1	-0.01244	-0.02697 to 0.00208	No	ns	0.111
WAK2cTAP vs. qua2-1/WAK2cTAP	-0.006111	-0.01984 to 0.00761	No	ns	0.6113
qua2-1 vs. qua2-1/WAK2cTAP	0.006333	-0.009438 to 0.0221	No	ns	0.6843

Fig 4C RQ

F (3, 8) = 691.9 P<0.0001

Tukey's multiple comparisons test	Mean Diff.	95.00% CI of diff.	Significant?	Summary	Adjusted P Value
WT vs. WAK2cTAP	-25.46	-27.53 to -23.39	Yes	****	<0.0001
WT vs. qua2-1/WAK2cTAP	-0.52	-2.591 to 1.551	No	ns	0.8508
WT vs. qua2-1/WAK2cTAP	-4.9	-6.971 to -2.829	Yes	***	0.0003
WAK2cTAP vs. qua2-1/WAK2cTAP	24.94	22.87 to 27.01	Yes	****	<0.0001
WAK2cTAP vs. qua2-1/WAK2cTAP	20.56	18.49 to 22.63	Yes	****	<0.0001
qua2-1/WAK2cTAP vs. qua2-1/WAK2cTAP	-4.38	-6.451 to -2.309	Yes	***	0.0006

Fig4C ROS

F (3, 20) = 510.9 P<0.0001

Tukey's multiple comparisons test	Mean Diff.	95.00% CI of diff.	Significant?	Summary	Adjusted P Value
WT vs. WAK2cTAP	-113966	-123387 to -104545	Yes	****	<0.0001
WT vs. qua2-1/WAK2cTAP	-745	-10166 to 8676	No	ns	0.996
WT vs. *qua2-1* WAK2cTAP	-48629	-58050 to -39208	Yes	****	<0.0001
WAK2cTAP vs. qua2-1/WAK2cTAP	113221	103800 to 122642	Yes	****	<0.0001
WAK2cTAP vs. *qua2-1* WAK2cTAP	65337	55916 to 74758	Yes	****	<0.0001
qua2-1/WAK2cTAP vs. *qua2-1* WAK2cTAP	-47884	-57305 to -38463	Yes	****	<0.0001

Fig 7A OG

F (3, 20) = 134.6 P<0.0001

Tukey's multiple comparisons test	Mean Diff.	95.00% CI of diff.	Significant?	Summary	Adjusted P Value
wakΔ 0 vs. wakΔOG	-40101	-106583 to 26381	No	ns	0.3555
wakΔ 0 vs. WT 0	-78143	-144625 to -11661	Yes	*	0.0177
wakΔ 0 vs. WT OG	-423909	-490391 to -357427	Yes	****	<0.0001
wakΔOG vs. WT 0	-38042	-104524 to 28440	No	ns	0.4002
wakΔOG vs. WT OG	-383808	-450290 to -317326	Yes	****	<0.0001
WT 0 vs. WT OG	-345766	-412248 to -279284	Yes	****	<0.0001

Fig 7A Chitin

F (3, 8) = 94.49 P<0.0001

Tukey's multiple comparisons test	Mean Diff.	95.00% CI of diff.	Significant?	Summary	Adjusted P Value
wakΔ0 vs. *wakΔ* chitin	-1314	-2708 to 80.36	No	ns	0.0649
wakΔ0 vs. WT 0	-1024	-2418 to 370.4	No	ns	0.165
wakΔ0 vs. WT chitin	-6658	-8052 to -5264	Yes	****	<0.0001
wakΔ chitin vs. WT 0	290	-1104 to 1684	No	ns	0.9069
wakΔ chitin vs. WT chitin	-5344	-6738 to -3950	Yes	****	<0.0001
WT 0 vs. WT chitin	-5634	-7028 to -4240	Yes	****	<0.0001

Fig 7 A Flg22

F (3, 8) = 277.3 P<0.0001

Tukey's multiple comparisons test	Mean Diff.	95.00% CI of diff.	Significant?	Summary	Adjusted P Value
wakΔ 0 vs. *wakΔ* Flg22	-186297	-253479 to -119115	Yes	****	<0.0001
wakΔ 0 vs. WT 0	-70516	-137698 to -3334	Yes	*	0.04
wakΔ 0 vs. WT Flg22	-555160	-622342 to -487978	Yes	****	<0.0001
wakΔ Flg22 vs. WT 0	115781	48599 to 182963	Yes	**	0.0025

continued

wakΔ Flg22 vs. WT Flg22	-368863	-436045 to -301681	Yes	****	<0.0001
WT 0 vs. WT Flg22	-484644	-551826 to -417462	Yes	****	<0.0001

Fig 7B FADlox

F (7, 16) = 56.68	P<0.0001				
Tukey's multiple comparisons test	Mean Diff.	95.00% CI of diff.	Significant?	Summary	Adjusted P Value
WT FAD vs. *WT OG FAD*	-395.5	-538.7 to -252.4	Yes	****	<0.0001
WT FAD vs. WT CH FAD	-657.1	-800.2 to -514.0	Yes	****	<0.0001
WT FAD vs. WT Fl FAD	-334.8	-477.9 to -191.6	Yes	****	<0.0001
WT FAD vs. *wak*Δ-/- FAD	-28.06	-171.2 to 115.1	No	ns	0.9965
WT FAD vs. wak$\Delta^{-/-}$ OG FAD	-469.4	-612.5 to -326.2	Yes	****	<0.0001
WT FAD vs. *wak*$\Delta^{-/-}$ CH FAD	-212	-355.1 to -68.84	Yes	**	0.002
WT FAD vs. wak$\Delta^{-/-}$ FL FAD	-269.8	-413.0 to -126.7	Yes	***	0.0001
WT OG FAD vs. WT CH FAD	-261.6	-404.7 to -118.5	Yes	***	0.0002
WT OG FAD vs. WT Fl FAD	60.76	-82.37 to 203.9	No	ns	0.812
WT OG FAD vs. *wak*Δ-/- FAD	367.5	224.3 to 510.6	Yes	****	<0.0001
WT OG FAD vs. wak$\Delta^{-/-}$ OG FAD	-73.84	-217.0 to 69.29	No	ns	0.637
WT OG FAD vs. *wak*$\Delta^{-/-}$ CH FAD	183.6	40.44 to 326.7	Yes	**	0.0076
WT OG FAD vs. wak$\Delta^{-/-}$ FL FAD	125.7	-17.42 to 268.8	No	ns	0.1077
WT CH FAD vs. WT Fl FAD	322.4	179.2 to 465.5	Yes	****	<0.0001
WT CH FAD vs. *wak*Δ-/- FAD	629.1	485.9 to 772.2	Yes	****	<0.0001
WT CH FAD vs. wak$\Delta^{-/-}$ OG FAD	187.8	44.63 to 330.9	Yes	**	0.0062
WT CH FAD vs. *wak*$\Delta^{-/-}$ CH FAD	445.2	302.0 to 588.3	Yes	****	<0.0001
WT CH FAD vs. wak$\Delta^{-/-}$ FL FAD	387.3	244.2 to 530.4	Yes	****	<0.0001
WT Fl FAD vs. *wak*Δ-/- FAD	306.7	163.6 to 449.8	Yes	****	<0.0001
WT Fl FAD vs. wak$\Delta^{-/-}$ OG FAD	-134.6	-277.7 to 8.530	No	ns	0.0732
WT Fl FAD vs. *wak*$\Delta^{-/-}$ CH FAD	122.8	-20.32 to 265.9	No	ns	0.1218
WT Fl FAD vs. wak$\Delta^{-/-}$ FL FAD	64.94	-78.18 to 208.1	No	ns	0.7599
*wak*Δ-/- FAD vs. wak$\Delta^{-/-}$ OG FAD	-441.3	-584.4 to -298.2	Yes	****	<0.0001
*wak*Δ-/- FAD vs. *wak*$\Delta^{-/-}$ CH FAD	-183.9	-327.0 to -40.78	Yes	**	0.0075
*wak*Δ-/- FAD vs. wak$\Delta^{-/-}$ FL FAD	-241.8	-384.9 to -98.65	Yes	***	0.0005
wak$\Delta^{-/-}$ OG FAD vs. *wak*$\Delta^{-/-}$ CH FAD	257.4	114.3 to 400.5	Yes	***	0.0003
wak$\Delta^{-/-}$ OG FAD vs. wak$\Delta^{-/-}$ FL FAD	199.5	56.41 to 342.7	Yes	**	0.0036
wak$\Delta^{-/-}$ CH FAD vs. wak$\Delta^{-/-}$ FL FAD	-57.86	-201.0 to 85.26	No	ns	0.8448

Fig7B WRKY53

F (7, 14) = 102.2	P<0.0001				
Tukey's multiple comparisons test	Mean Diff.	95.00% CI of diff.	Significant?	Summary	Adjusted P Value
WT WRKY54 vs. WT OG WRKY54	-0.367	-11.02 to 10.29	No	ns	>0.9999
WT WRKY54 vs. WT CH WRKY54	-27.03	-37.69 to -16.38	Yes	****	<0.0001
WT WRKY54 vs. WT Fl WRKY54	-36.75	-47.40 to -26.09	Yes	****	<0.0001
WT WRKY54 vs. *wak*$\Delta^{-/-}$ WRKY54	-0.8452	-11.50 to 9.809	No	ns	>0.9999
WT WRKY54 vs. *wak*$\Delta^{-/-}$ OG WRKY54	-0.6667	-11.32 to 9.988	No	ns	>0.9999
WT WRKY54 vs. *wak*$\Delta^{-/-}$ CH WRKY54	-26.66	-37.31 to -16.01	Yes	****	<0.0001
WT WRKY54 vs. *wak*$\Delta^{-/-}$ FL WRKY54	-29.15	-39.80 to -18.50	Yes	****	<0.0001
WT OG WRKY54 vs. WT CH WRKY54	-26.66	-34.20 to -19.13	Yes	****	<0.0001
WT OG WRKY54 vs. WT Fl WRKY54	-36.38	-43.91 to -28.85	Yes	****	<0.0001
WT OG WRKY54 vs. *wak*$\Delta^{-/-}$ WRKY54	-0.4782	-8.012 to 7.056	No	ns	>0.9999
WT OG WRKY54 vs. *wak*$\Delta^{-/-}$ OG WRKY54	-0.2997	-7.834 to 7.234	No	ns	>0.9999
WT OG WRKY54 vs. *wak*$\Delta^{-/-}$ CH WRKY54	-26.29	-33.83 to -18.76	Yes	****	<0.0001
WT OG WRKY54 vs. *wak*$\Delta^{-/-}$ FL WRKY54	-28.78	-36.32 to -21.25	Yes	****	<0.0001
WT CH WRKY54 vs. WT Fl WRKY54	-9.717	-17.25 to -2.183	Yes	**	0.008
WT CH WRKY54 vs. *wak*$\Delta^{-/-}$ WRKY54	26.19	18.65 to 33.72	Yes	****	<0.0001
WT CH WRKY54 vs. *wak*$\Delta^{-/-}$ OG WRKY54	26.36	18.83 to 33.90	Yes	****	<0.0001
WT CH WRKY54 vs. *wak*$\Delta^{-/-}$ CH WRKY54	0.3711	-7.163 to 7.905	No	ns	>0.9999
WT CH WRKY54 vs. *wak*$\Delta^{-/-}$ FL WRKY54	-2.119	-9.653 to 5.415	No	ns	0.9682
WT Fl WRKY54 vs. *wak*$\Delta^{-/-}$ WRKY54	35.9	28.37 to 43.44	Yes	****	<0.0001
WT Fl WRKY54 vs. *wak*$\Delta^{-/-}$ OG WRKY54	36.08	28.55 to 43.62	Yes	****	<0.0001

continued

WT Fl WRKY54 vs. *wak*$\Delta^{-/-}$ CH WRKY54	10.09	2.554 to 17.62	Yes	**	0.0058
WT Fl WRKY54 vs. *wak*$\Delta^{-/-}$ FL WRKY54	7.598	0.06390 to 15.13	Yes	*	0.0474
wak$\Delta^{-/-}$ WRKY54 vs. *wak*$\Delta^{-/-}$ OG WRKY54	0.1785	-7.355 to 7.712	No	ns	>0.9999
wak$\Delta^{-/-}$ WRKY54 vs. *wak*$\Delta^{-/-}$ CH WRKY54	-25.81	-33.35 to -18.28	Yes	****	<0.0001
wak$\Delta^{-/-}$ WRKY54 vs. *wak*$\Delta^{-/-}$ FL WRKY54	-28.3	-35.84 to -20.77	Yes	****	<0.0001
wak$\Delta^{-/-}$ OG WRKY54 vs. *wak*$\Delta^{-/-}$ CH WRKY54	-25.99	-33.53 to -18.46	Yes	****	<0.0001
wak$\Delta^{-/-}$ OG WRKY54 vs. *wak*$\Delta^{-/-}$ FL WRKY54	-28.48	-36.02 to -20.95	Yes	****	<0.0001
wak$\Delta^{-/-}$ CH WRKY54 vs. *wak*$\Delta^{-/-}$ FL WRKY54	-2.49	-10.02 to 5.044	No	ns	0.9291

Fig S5

$F (7, 40) = 20.07$ P<0.0001

Tukey's multiple comparisons test	Mean Diff.	95.00% CI of diff.	Significant?	Summary	Adjusted P Value
WT O vs. WT OG	-155610	-259951 to -51269	Yes	***	0.0006
WT O vs. *esmd*	53051	-51290 to 157392	No	ns	0.7327
WT O vs. *esmd* OG	-211185	-315526 to -106844	Yes	****	<0.0001
WT O vs. CTAP	-125585	-229926 to -21244	Yes	**	0.0091
WT O vs. CTAP OG	-127112	-231453 to -22771	Yes	**	0.008
WT O vs. *esmd* CTAP	45046	-59295 to 149387	No	ns	0.8608
WT O vs. *esmd* CTAPOG	-170703	-275044 to -66362	Yes	***	0.0001
WT OG vs. *esmd*	208661	104320 to 313002	Yes	****	<0.0001
WT OG vs. *esmd* OG	-55575	-159916 to 48766	No	ns	0.6856
WT OG vs. CTAP	30025	-74316 to 134366	No	ns	0.9823
WT OG vs. CTAP OG	28498	-75843 to 132839	No	ns	0.9869
WT OG vs. *esmd* CTAP	200656	96315 to 304997	Yes	****	<0.0001
WT OG vs. *esmd* CTAPOG	-15093	-119434 to 89248	No	ns	0.9998
esmd vs. *esmd* OG	-264236	-368577 to -159895	Yes	****	<0.0001
esmd vs. CTAP	-178636	-282977 to -74295	Yes	****	<0.0001
esmd vs. CTAP OG	-180163	-284504 to -75822	Yes	****	<0.0001
esmd vs. *esmd* CTAP	-8005	-112346 to 96336	No	ns	>0.9999
esmd vs. *esmd* CTAPOG	-223754	-328095 to -119413	Yes	****	<0.0001
esmd OG vs. CTAP	85600	-18741 to 189941	No	ns	0.178
esmd OG vs. CTAP OG	84073	-20268 to 188414	No	ns	0.195
esmd OG vs. *esmd* CTAP	256231	151890 to 360572	Yes	****	<0.0001
esmd OG vs. *esmd* CTAPOG	40482	-63859 to 144823	No	ns	0.9146
CTAP vs. CTAP OG	-1527	-105868 to 102814	No	ns	>0.9999
CTAP vs. *esmd* CTAP	170631	66290 to 274972	Yes	***	0.0001
CTAP vs. *esmd* CTAPOG	-45118	-149459 to 59223	No	ns	0.8599
CTAP OG vs. *esmd* CTAP	172158	67817 to 276499	Yes	***	0.0001
CTAP OG vs. *esmd* CTAPOG	-43591	-147932 to 60750	No	ns	0.8797
esmd CTAP vs. *esmd* CTAPOG	-215749	-320090 to -111408	Yes	****	<0.0001
