# Supplementary material for: Effects of Arabidopsis wall associated kinase mutations on ESMERALDA1 and elicitor induced ROS
Source: PLoS One. 2021 May 20;16(5):e0251922. doi: 10.1371/journal.pone.0251922 (PMC8136723; doi:10.1371/journal.pone.0251922)
Supplement: S3 Fig — A. Top is shown the consensus SgRNA, and the annealed pairs selected for WAK4 in green, and WAK2 in mustard. Red bases indicate the required NGG. The result of the Sg cut and fused WAK4-2 sequence is shown at the bottom. B. Root length in mm of WT and wakΔ seedlings grown on MS agar. C. List of oligonucleotides used in the analysis. (PDF) [file pone.0251922.s003.pdf]

A

GN19NGG Sg RNAs

WAK4 Sg RNA
gattGCAA TCCTGAATAC GTTGAA3’ WAK4 CRISPRF
CGTT AGGACTTATG CAACTT caaa 5’ WAK4CRISPRR

WAK4 seq
1601 CACCACTAAT ACCTGCAAAC CTAAAGGCAA TCCTGAATAC GTTGAATGGA CTACAATTGT

WAK2 SG RNA
5’ gatt GGG AATCCATACC TTCCAAA WAK2CRISPF
CCC TTAGGTATGG AAGGTTT caaa 5’ WAK2CRISPR

WAK2 seq
851 GTTTAGAAGG TTTTGAGGGG AATCCATACC TTCCAAACGG TTGTCAAGGTACTTTAACTTG

WAKΔ seq
CTAATACCTGCAAACCTAAAGGCAATCCTGAATACGTTCAAACGGTTGTCAAGGTACTTTAACTTG

B

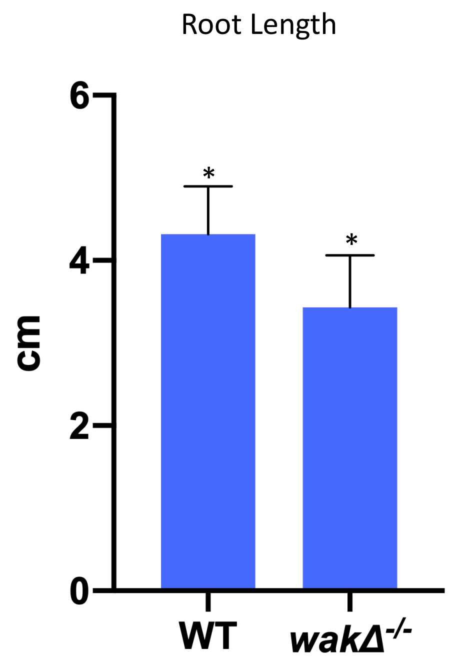

Fig S3C

wak23UTR: CCAAGTAAAGGGAAATATGAACATGATTATTGATGATGTAC
wak45UTF: CTGCGTAAAGATCAAGTTTGGAGAGAAAATAAATAAAGAAGATG
wak1NF2: GCTTGGCGTTGCCTGTATACAACAG
wak1Rspecific: CAGGAGATGAAGAATTTAGGAACCAGGTTCATG
wak2UF: GAGAGAAGATGAAGGTACAGGAGGGTTTGTTCGTGGTGG
wakTMR: GAGCTCGTTCTTCCGGTGCTTATTT

QUA2 F:5’-CAGGGATCTTAGATTTATAGCAGCAAC-3’
QUA2R: 5’-GAAACCGAACCGGAAACATA-3’
ESMD1F: 5’-GGCGATAGGTTTCAATGATGAATTAAG-3’
ESMD1R: 5’-CCAAGTAAAGGGAAATATGAACATGATTATTG ATGATGTAC-3’
