# Supplementary material for: Effects of Arabidopsis wall associated kinase mutations on ESMERALDA1 and elicitor induced ROS
Source: PLoS One. 2021 May 20;16(5):e0251922. doi: 10.1371/journal.pone.0251922 (PMC8136723; doi:10.1371/journal.pone.0251922)
Supplement: S4 Fig — No other significant changes (padj<0.05) in the transcripts were detected. Red highlights the WAK genes, and green other genes. (PDF) [file pone.0251922.s004.pdf]

	**readcount** wakΔ	**readcount** WT	log fold change	pval	padj	Gene
AT1G21210	5.38708381	3.8100569	0.49969	0.85501	1	WAK4
AT1G21220	0	0.29493682		1	1	
AT1G21230	0	0				WAK5
AT1G21240	0	9.80530005		0.00019	0.4031	WAK3
AT1G21245	0	0				
AT1G21250	0	392.3822		1.98E-13	1.14E-09	WAK1
AT1G21260	0	52.3114151		1.16E-19	1.33E-15	
AT1G21270	4.1232773	1608.53113	-8.6077	1.97E-69	4.52E-65	WAK2
AT1G05675	147.340294	54.4294962	1.4367	3.51E-07	0.0013412	UGT74E1
AT2G14560	242.314104	116.72578	1.0538	1.42E-05	0.046702	LURP1
AT3G30720	199.21816	43.1634462	2.2065	4.20E-16	3.21E-12	QQS
AT5G65080	122.272012	8.37794837	3.8674	7.47E-12	3.43E-08	MAF5
