# Supplementary figures and images for: Effects of Arabidopsis wall associated kinase mutations on ESMERALDA1 and elicitor induced ROS
Source: PLoS One. 2021 May 20;16(5):e0251922. doi: 10.1371/journal.pone.0251922 (PMC8136723; doi:10.1371/journal.pone.0251922)

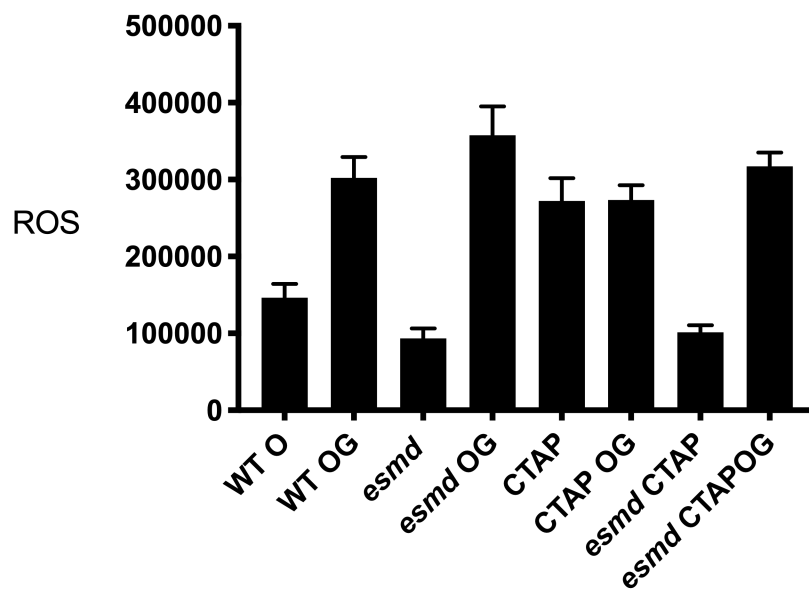

Supplement: S5 Fig — ANOVA and Tukey’s tests are reported in the text and S2 Fig. (PDF) [file pone.0251922.s005.pdf]

Fig 1

vs TAP

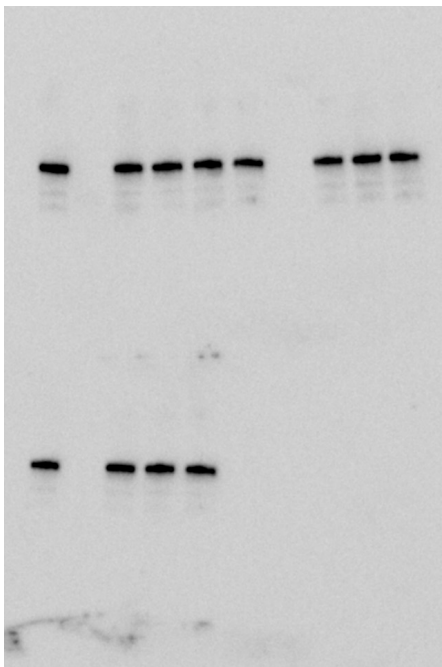

vs TUB

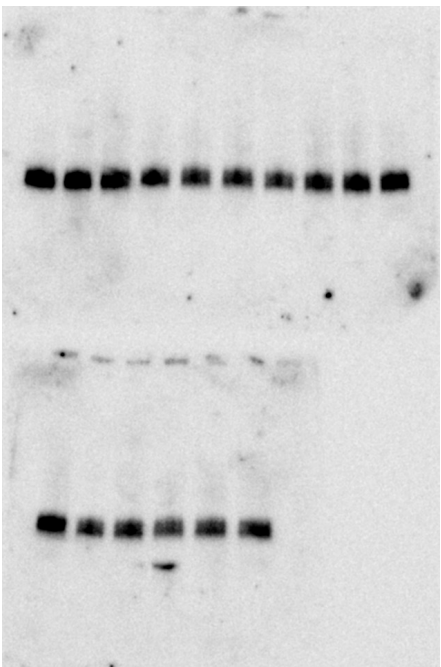

Fig 2

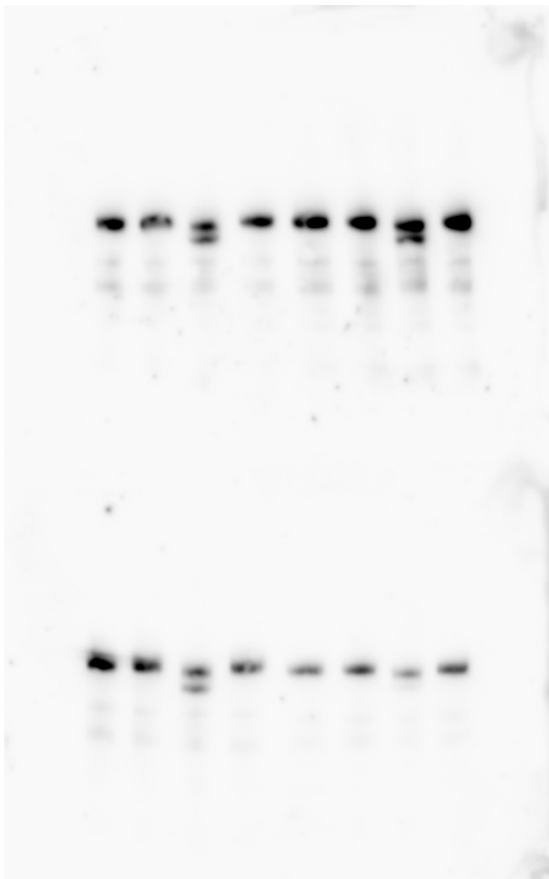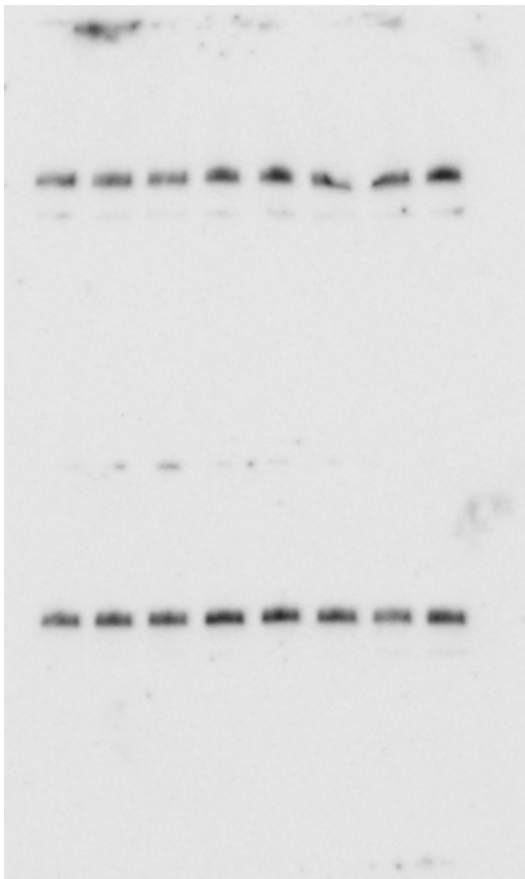

Supplement: S6 Fig — (PDF) [file pone.0251922.s006.pdf]
